# Machine Learning Improves Risk Stratification in Myelofibrosis: An Analysis of the Spanish Registry of Myelofibrosis

**DOI:** 10.1097/HS9.0000000000000818

**Published:** 2022-12-20

**Authors:** Adrián Mosquera-Orgueira, Manuel Pérez-Encinas, Alberto Hernández-Sánchez, Teresa González-Martínez, Eduardo Arellano-Rodrigo, Javier Martínez-Elicegui, Ángela Villaverde-Ramiro, José-María Raya, Rosa Ayala, Francisca Ferrer-Marín, María-Laura Fox, Patricia Velez, Elvira Mora, Blanca Xicoy, María-Isabel Mata-Vázquez, María García-Fortes, Anna Angona, Beatriz Cuevas, María-Alicia Senín, Angel Ramírez-Payer, María-José Ramírez, Raúl Pérez-López, Sonia González de Villambrosía, Clara Martínez-Valverde, María-Teresa Gómez-Casares, Carmen García-Hernández, Mercedes Gasior, Beatriz Bellosillo, Juan-Luis Steegmann, Alberto Álvarez-Larrán, Jesús María Hernández-Rivas, Juan Carlos Hernández-Boluda

**Affiliations:** 1 Hospital Clínico Universitario, Santiago de Compostela, Spain; 2 Hospital Clínico, Salamanca, Spain; 3 Hospital Clínic, Institut d’Investigacions Biomèdiques August Pi i Sunyer, Barcelona, Spain; 4 Hospital Universitario de Canarias, Tenerife, Spain; 5 Hospital Universitario 12 de Octubre, Madrid, Spain; 6 Hospital Morales Meseguer, Universidad Católica San Antonio de Murcia, Centro de Investigación Biomédica en Red de Enfermedades Raras, Murcia, Spain; 7 Vall d’Hebron Institute of Oncology (VHIO), Vall d’Hebron Hospital Universitari, Vall d’Hebron Barcelona Hospital Campus, Spain; 8 Hospital del Mar, Barcelona, Spain; 9 Hospital Universitario La Fe, Valencia, Spain; 10 Hospital Germans Trias i Pujol, Institut Català d’Oncologia, Josep Carreras Leukemia Research Institute, Universitat Autònoma de Barcelona, Badalona, Spain; 11 Hospital Costa del Sol, Marbella, Spain; 12 Hospital Virgen de la Victoria, Málaga, Spain; 13 Hospital Josep Trueta, Institut Catalá d’Oncologia, Girona, Spain; 14 Hospital Universitario de Burgos, Burgos, Spain; 15 Institut Catalá d’Oncologia, L’Hospitalet de Llobregat, Spain; 16 Hospital Universitario Central de Asturias, Oviedo, Spain; 17 Hospital General, Jerez de la Frontera, Spain; 18 Hospital Virgen de la Arrixaca, Murcia, Spain; 19 Hospital Marqués de Valdecilla, Santander, Spain; 20 Hospital de la Santa Creu i Sant Pau, Barcelona, Spain; 21 Hospital Dr Negrín, Las Palmas de Gran Canaria, Spain; 22 Hospital General, Alicante, Spain; 23 Hospital La Paz, Madrid, Spain; 24 Hospital de La Princesa, Madrid, Spain; 25 Hospital Clínico Universitario-INCLIVA, Valencia, Spain

## Abstract

Myelofibrosis (MF) is a myeloproliferative neoplasm (MPN) with heterogeneous clinical course. Allogeneic hematopoietic cell transplantation remains the only curative therapy, but its morbidity and mortality require careful candidate selection. Therefore, accurate disease risk prognostication is critical for treatment decision-making. We obtained registry data from patients diagnosed with MF in 60 Spanish institutions (N = 1386). These were randomly divided into a training set (80%) and a test set (20%). A machine learning (ML) technique (random forest) was used to model overall survival (OS) and leukemia-free survival (LFS) in the training set, and the results were validated in the test set. We derived the AIPSS-MF (Artificial Intelligence Prognostic Scoring System for Myelofibrosis) model, which was based on 8 clinical variables at diagnosis and achieved high accuracy in predicting OS (training set c-index, 0.750; test set c-index, 0.744) and LFS (training set c-index, 0.697; test set c-index, 0.703). No improvement was obtained with the inclusion of MPN driver mutations in the model. We were unable to adequately assess the potential benefit of including adverse cytogenetics or high-risk mutations due to the lack of these data in many patients. AIPSS-MF was superior to the IPSS regardless of MF subtype and age range and outperformed the MYSEC-PM in patients with secondary MF. In conclusion, we have developed a prediction model based exclusively on clinical variables that provides individualized prognostic estimates in patients with primary and secondary MF. The use of AIPSS-MF in combination with predictive models that incorporate genetic information may improve disease risk stratification.

## INTRODUCTION

Myelofibrosis (MF) is a rare chronic myeloproliferative neoplasm (MPN) that appears de novo (primary myelofibrosis [PMF]), or after previous polycythemia vera (PV) or essential thrombocythemia (ET) (secondary myelofibrosis [SMF]).^1^ Constitutive signaling through the JAK-STAT pathway via activating mutations of *JAK2*, *CALR*, and *MPL* genes plays a key role in its pathogenesis, while concomitant somatic mutations, mostly affecting epigenetic modifiers or spliceosome components, may influence clinical phenotype or promote disease progression.^2,3^

The median survival of MF patients is about 6 years, but there is great individual variability, with the main causes of death being leukemic transformation (20% of cases), disease progression and infections.^4,5^ Management of MF is challenging because there are no disease-modifying drugs,^6,7^ and the only curative treatment is allogeneic hematopoietic cell transplantation (allo-HCT),^8–10^ which is associated with significant morbidity and mortality. Therefore, the risks of allo-HCT must be weighed up against expected survival without transplantation in each patient. According to consensus recommendations of the NCCN, ELN, and EBMT/ELN,^8–10^ allo-HCT should be considered in eligible MF patients with an expected survival below 5 years. Consequently, accurate prediction of survival is critical for transplant decision-making.^11^

Conventional prognostic models in use are the International Prognostic Scoring System (IPSS),^12^ which should be used at diagnosis, and the Dynamic IPSS (DIPSS) and DIPSS plus,^13,14^ applicable at any time during the clinical course. These models were derived from patients with PMF, so a scoring system named the Myelofibrosis Secondary to PV and ET-Prognostic Model (MYSEC-PM) was subsequently developed to improve risk stratification in patients with SMF.^15^

Including cytogenetic and molecular data in contemporary stratification models has led to more comprehensive prognostication. In this respect, the Molecular enhanced International Prognostic Score System (MIPSS70) is based on clinical, histological, and molecular factors,^16^ while the MIPSS70+ v2.0 includes clinical, cytogenetic, and molecular risk factors,^17^ and the Genetically Inspired Prognostic Scoring System (GIPSS) is restricted to genomic and cytogenetic data only.^18^ Finally, the prognostic calculator developed by Grinfeld et al^19^ differ in that rather than discriminating risk groups, it makes an individual prediction of survival and risk of acute myeloid leukemia (AML) based on clinical characteristics, cytogenetics, and comprehensive molecular data. However, the main limitation to the general applicability of these models in clinical practice is the need for cytogenetic studies, which in many cases cannot be performed on bone marrow samples due to failure to obtain marrow (dry tap),^12,20,21^ and the need for next-generation sequencing (NGS). Therefore, conventional risk models based primarily on clinical variables are still widely used in the clinical setting.^10,11,22–24^

The recent development of machine learning (ML) in medicine has become key to overcoming some of the limitations of classical prognostic scores.^25^ ML is a field of artificial intelligence in which prediction is based on modeling of outcomes considering the complex interactions between multiple variables derived from real examples, rather than the application of human-made rules. In MF, these advanced techniques can provide personalized survival predictions based on the clinical experience of thousands of patients. With this in mind, we aimed to develop a new model for MF risk stratification using basic clinical information obtained at disease diagnosis.

## MATERIALS AND METHODS

### Data source

We retrieved original data included in the Spanish Registry of Myelofibrosis from 1617 patients diagnosed with MF between January 2000 and October 2021 in 60 centers. This is a nationwide registry (GEM-MIE-2014-01) contributed by centers affiliated to the *Grupo Español de enfermedades Mieloproliferativas Filadelfia Negativas* (GEMFIN). Informed consent for inclusion in the registry was obtained from all patients. In every patient, MF diagnosis was made according to the World Health Organization (WHO) criteria in use at the time of first observation. The study was approved by the GEMFIN scientific board and was conducted in accordance with the Declaration of Helsinki.

Variables with a low level of missing data (<15%) were selected for the analyses. The selected variables were: age at MF diagnosis, sex, MF subtype, palpable splenomegaly or hepatomegaly, splenomegaly-related symptoms, constitutional symptoms, hemoglobin levels, platelet count, leukocyte count, monocyte count, circulating blast cells, and leukoerytroblastosis (ie, presence of nucleated red cells, immature granulocytes and dacryocytes in peripheral blood). After filtering out cases with incomplete annotations, a total of 1386 patients remained for analysis (tier 1 patients, missing data rate 2.76%). These patients were randomly divided into a training set (80% of the cohort, N = 1109) and a test set (20% of the cohort, N = 277). Two hundred thirty-one patients with a high rate of incomplete annotations in these variables (30.98%) were not included in the initial analysis (tier 2 patients).

### Main study outcomes

Overall survival (OS) was defined as time from MF diagnosis to death from any cause. In some analyses, for comparison with other prognostic models, survival was censored at the time of allo-HCT. Leukemia-free survival (LFS) was defined as time from MF diagnosis to date of leukemic transformation (uncensored) or last contact/date of death (censored).

### Variable selection and model development

Univariate Cox regression (*survival* package)^26^ was used to evaluate the association of each variable with OS in the training set. Variables with a *P* value <0.01 were selected for a multivariate model using random forests (*randomForestSRC* package).^27^ Missing variables were imputed in each cohort separately using a missing data algorithm developed by Ishwaran et al.^27^ Random forests were created with 1000 trees. For cross-validation, sampling was performed without replacement, which by default takes 0.632 times the sample size. Predictions were cross-validated in the training set and then validated in the test cohort. This was done to rule out overfitting of performance metrics in the training set related to either variable selection or the imputation process. The discriminative capacity of the random forest models in the training set was evaluated with out-of-bag estimates of the concordance index (c-index).

The precision of the different predictors was assessed using time-dependent areas under the curve (AUCs) derived from Cox survival models.^28^ For these calculations, cross-validation was performed with the *bootcv* algorithm using 500 cycles. In each cycle, 75% of samples were used for training and 25% for testing. The c-indexes of these Cox models were computed with bootstrapping in both the training and test set (500 cycles).^29^ In the particular case of the training set, all random forest predictions used as input for downstream analysis were out-of-bag to reduce the risk of overfitting during the training phase of the model.

### Mutational analysis

DNA from bone marrow or peripheral blood was used for mutation analysis. MPN driver mutations were detected using standard methods.^30,31^ NGS was used to detect somatic mutations involved in myeloid malignancies. Only pathogenic or probably pathogenic mutations with a variant allele frequency (VAF) *≥* 5% were considered. High molecular risk was defined by the presence of mutations in *ASXL1*, *EZH2*, *IDH1*, *IDH2*, *SRSF2* genes or by the presence of the *U2AF1*^*Q157*^ mutation.^17^

## RESULTS

### Study population, clinical outcomes, and variable selection

**Figure 1.**
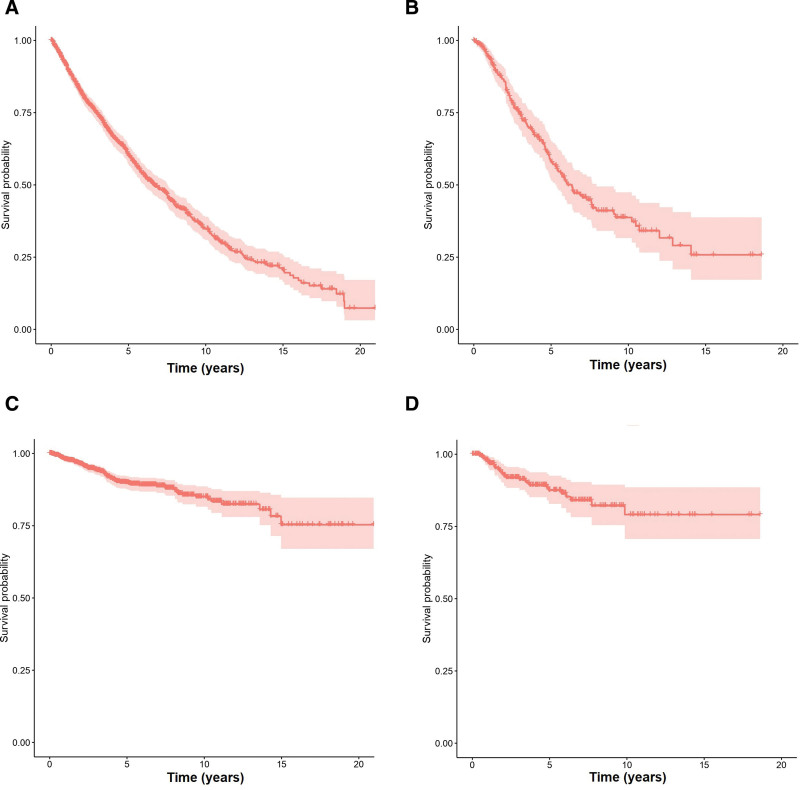
**(A and B) Kaplan–Meier plots representing overall survival for the training and test sets, respectively.** (C and D) Kaplan–Meier plots representing leukemia-free survival for the training and test sets, respectively.

Baseline characteristics and main clinical outcomes of patients included in each cohort are shown in Table 1 and Figure 1. Eight variables were associated with OS with a *P* value *≤*0.01 in the training set (Table 2). These variables were age at MF diagnosis, sex, hemoglobin level, percentage of blasts in peripheral blood, leukocyte count, platelet count, leukoerytroblastosis in peripheral blood, and the presence of constitutional symptoms.

**Table 1:** Baseline Characteristics of Patients in the Training and Test Sets

Variable	Training	Test
N	1109	277
Age^a^, y	68.79 (18.65–94.25)	68.77 (23.07–91.67)
Age > 65 y, %	61.77	59.93
Male/female sex, %	57.71/42.29	59.93/40.07
Primary myelofibrosis, %	59.51	63.90
ECOG performance status >1, %	12.62	14.06
Constitutional symptoms, %	42.20	38.27
Palpable splenomegaly, %	75.95	75.64
Palpable hepatomegaly, %	18.13	20.53
Leukocyte counts^a^, ×10^9^/L	9.88 (0.90–101.10)	10.50 (0.67–104.00)
Leukocytes >25 × 10^9^/L, %	10.55	10.62
Hemoglobin level^a^, g/dL	10.9 (3.2–19.4)	10.8 (4.4–18.7)
Hemoglobin < 10 g/dL, %	33.12	32.84
Platelet count^a^, ×10^9^/L	316 (5–2091)	309 (14–1640)
Platelets < 100 × 10^9^/L, %	12.59	16.11
Blood blasts count^b^, %	0.94 (0–15)	1.02 (0–11)
Blood blasts ≥1%, %	39.5	42.11
Leukoerythroblastosis, %	60.8	58.2
Serum LDH levels^a^, IU/L	638 (68–4037)	673 (180–2374)
WHO bone marrow fibrosis grade 0-1, %	14.05	16.53
WHO bone marrow fibrosis grade 2-3, %	85.95	83.47
JAK2-mutated, %	59.69	57.40
CALR-mutated, %	15.42	13.36
MPL-mutated, %	3.33	2.89
Triple negative, %	3.88	7.58
Genotype not fully annotated, %	17.67	18.77
IPSS low risk, %	11.09	11.19
IPSS intermediate-1, %	23.25	23.47
IPSS intermediate-2, %	26.42	27.80
IPSS high risk, %	21.28	18.77
Median follow-up, y	6.56	6.51
JAK inhibitor treatment, %	38.95	37.54
Allogeneic hematopoietic cell transplantation, %	9.64	10.47
Median overall survival (95% CI), y	6.64 (6.06–7.55)	6.35 (5.35–7.93)
Progression to AML, %	8.30	10.47

aMedian (range).

bMean (range).

AML = acute myeloid leukemia; ECOG = Eastern Cooperative Oncology Group; LDH = lactate dehydrogenase; IPSS = International Prognostic Scoring System; LDH; WHO = World Health Organization.

**Table 2:** Variable Association With Overall Survival in the Training Set (Cox Regression)

Variable	HR	*P* value
Age at diagnosis	1.06	<0.0001
Sex	1.78	<0.0001
PMF vs PET-MF	0.81	0.05
PMF vs PPV-MF	1.03	0.83
Palpable splenomegaly	1.25	0.05
Palpable hepatomegaly	1.10	0.38
Symptomatic splenomegaly	1.16	0.17
Constitutional symptoms	1.44	<0.0001
Hemoglobin level	0.98	<0.0001
Leukocyte count	1.03	<0.0001
Monocyte count	1	0.14
Platelet count	0.99	<0.0001
Blasts in peripheral blood (%)	1.22	<0.0001
Leukoerythroblastosis	1.50	<0.0001

PET-MF = postessential thrombocythemia myelofibrosis; PMF = primary myelofibrosis; PPV-MF = postpolycythemia vera myelofibrosis.

### Prediction of survival and AML transformation

**Figure 2.**
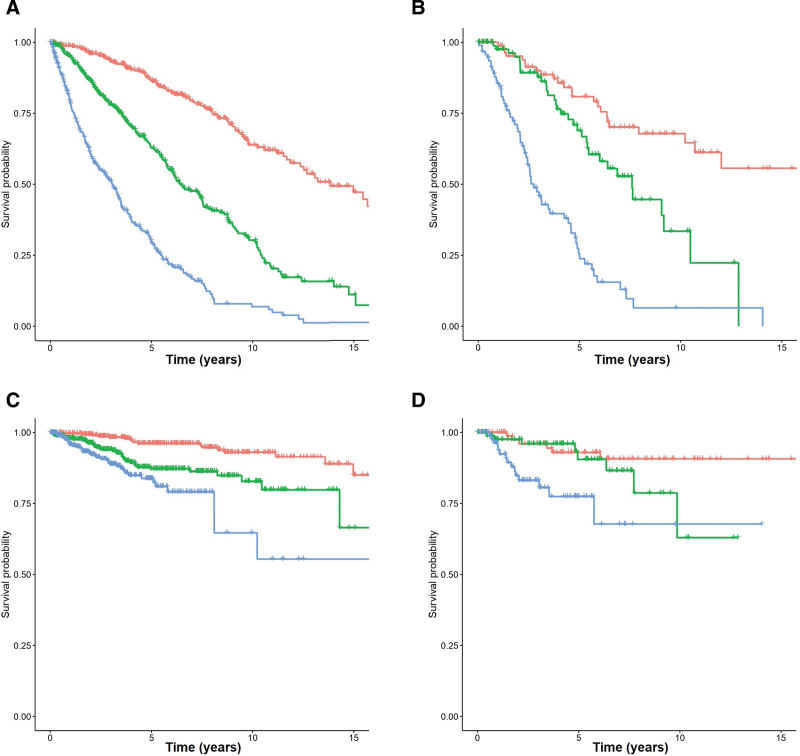
**Survival according to the ML model.** Patients were split according to the predicted tertile of overall survival according to the model. Each branch represents a tertile of patients with either low-risk (red), intermediate-risk (green), or high-risk (blue) disease. (A and B) The association with overall survival for the training and test cohorts, respectively, and (C and D) the association with time to leukemic transformation in the training and test sets, respectively. ML = machine learning.

A random forest model was created to predict OS with the significant variables in the training set. This model achieved an out-of-bag c-index of 0.750 in the training set and 0.744 in the test set (Figure 2). For comparison, the c-indexes of the IPSS predictor using random forests were 0.599 and 0.685 in the training and test cohorts, respectively. We also evaluated the c-indexes using traditional Cox regression to rule out underperformance of random forests with the IPSS scores, using the ML model predictions as input. Bootstrapped c-indexes were similar for the ML model (0.730 and 0.738 in the training and test sets) and substantially superior to those of the IPSS (c-indexes of 0.664 and 0.674 in the training and test sets). We also evaluated the performance of the IPSS-related variables as input to the random forests model, revealing a better performance than the IPSS (c-indexes 0.704 and 0.718), but still inferior to the new model.

Importantly, the model performed equally well in patients diagnosed before and after 2012, when ruxolitinib became available in Spain for the treatment of MF patients. In the training set, the bootstrapped c-index of the ML model in the training set was 0.723 and 0.742 for patients diagnosed before and after 2012. These results were superior to those of the IPSS (c-indexes; 0.674 and 0.680, respectively). In the test set, the c-indexes of the ML model were 0.750 and 0.730 for patients diagnosed before and after 2012, whereas the c-index of the IPSS was 0.686 and 0.660. Additionally, we evaluated the performance of the model among patients who were subsequently treated with any JAK inhibitor. Bootstrapped c-indexes for the ML model and the IPSS score among patients treated with JAK inhibitors were 0.700 and 0.650 in the training set; and 0.736 and 0.689 in the test set.

**Figure 3.**
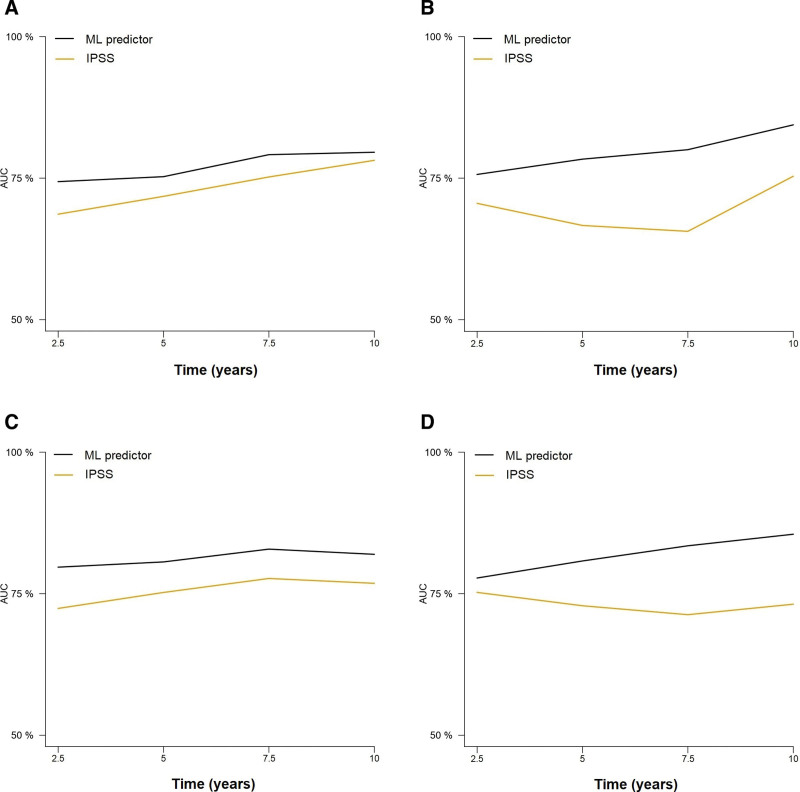
**Time-dependent cross-validated AUCs in the training (A) and test (B) cohorts for the prediction of leukemia-free survival according to the ML model and the IPSS score.** Time-dependent cross-validated AUCs in the training (C) and test cohorts (D) for predicting overall survival according to the ML model and the IPSS score. AUCs = areas under the curve; IPSS = International Prognostic Scoring System, ML = machine learning.

We then evaluated the capacity of the model to predict LFS. Initially, we used the same OS predictors created by the ML algorithm to model the time to AML transformation using Cox regression. C-indexes after bootstrapping were 0.697 and 0.703 in the training and test set, respectively, indicating that the same model retains substantial predictive ability for leukemic transformation. Time-dependent cross-validated AUCs also indicate that these predictions achieved high accuracy (Figure 3A and B; Suppl. Table S1). A random forest model based on the raw values of the 8 variables achieved similar accuracy in both cohorts (c-indexes; 0.702 and 0.676 in the training and test sets, respectively).

### Comparison of the ML model with the IPSS for predicting OS

**Figure 4.**
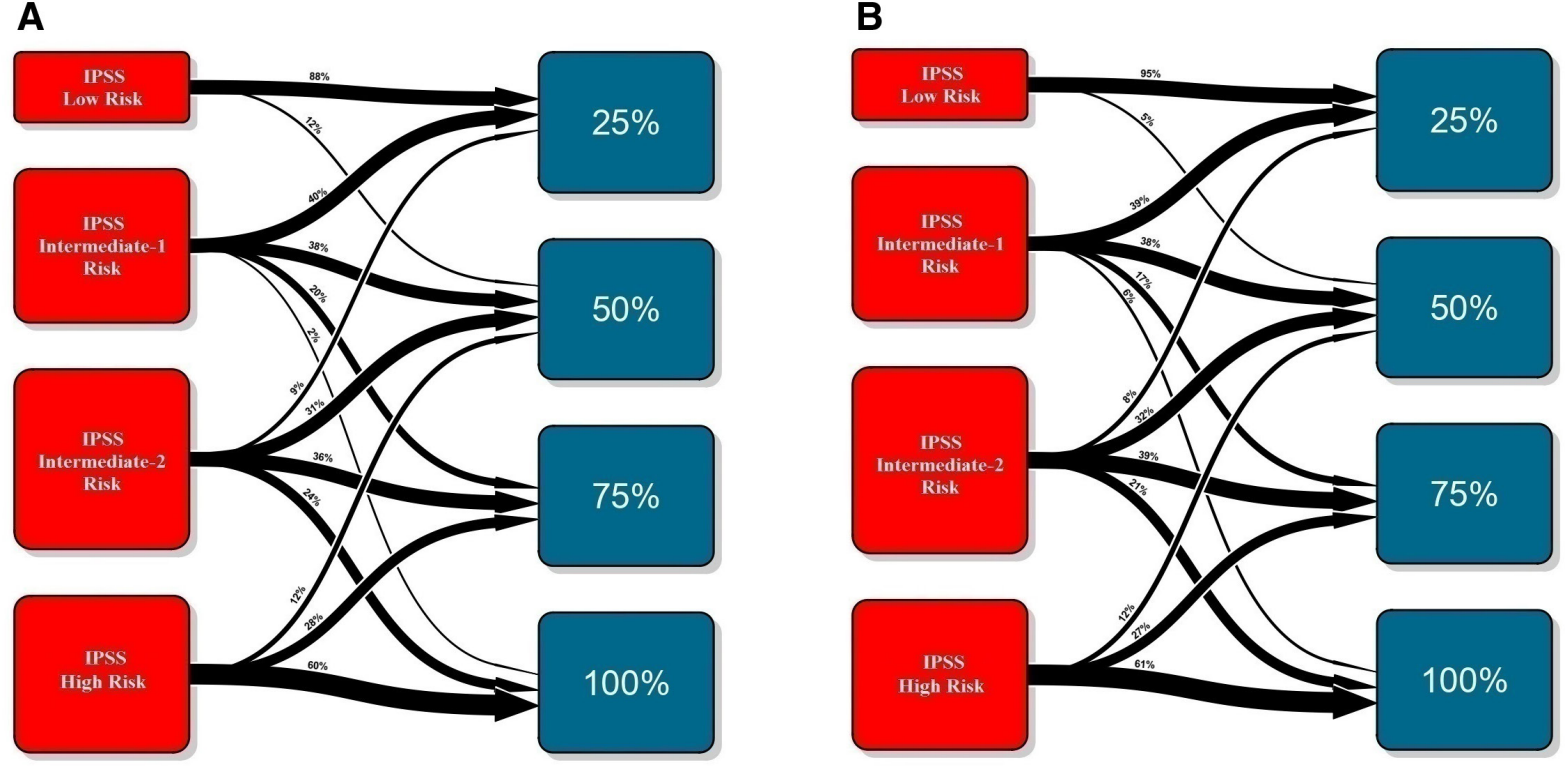
**Transition plots representing the flow of patients between the different IPSS groups to the different survival quartiles predicted by the AIPSS-MF model in the training (A) and test (B) cohorts.** However, it is important to note that the AIPSS-MF does not assign patients into risk groups but provides individual predictions of overall and leukemia-free survival. AIPSS-MF = Artificial Intelligence Prognostic Scoring System for Myelofibrosis; IPSS = International Prognostic Scoring System.

**Figure 5.**
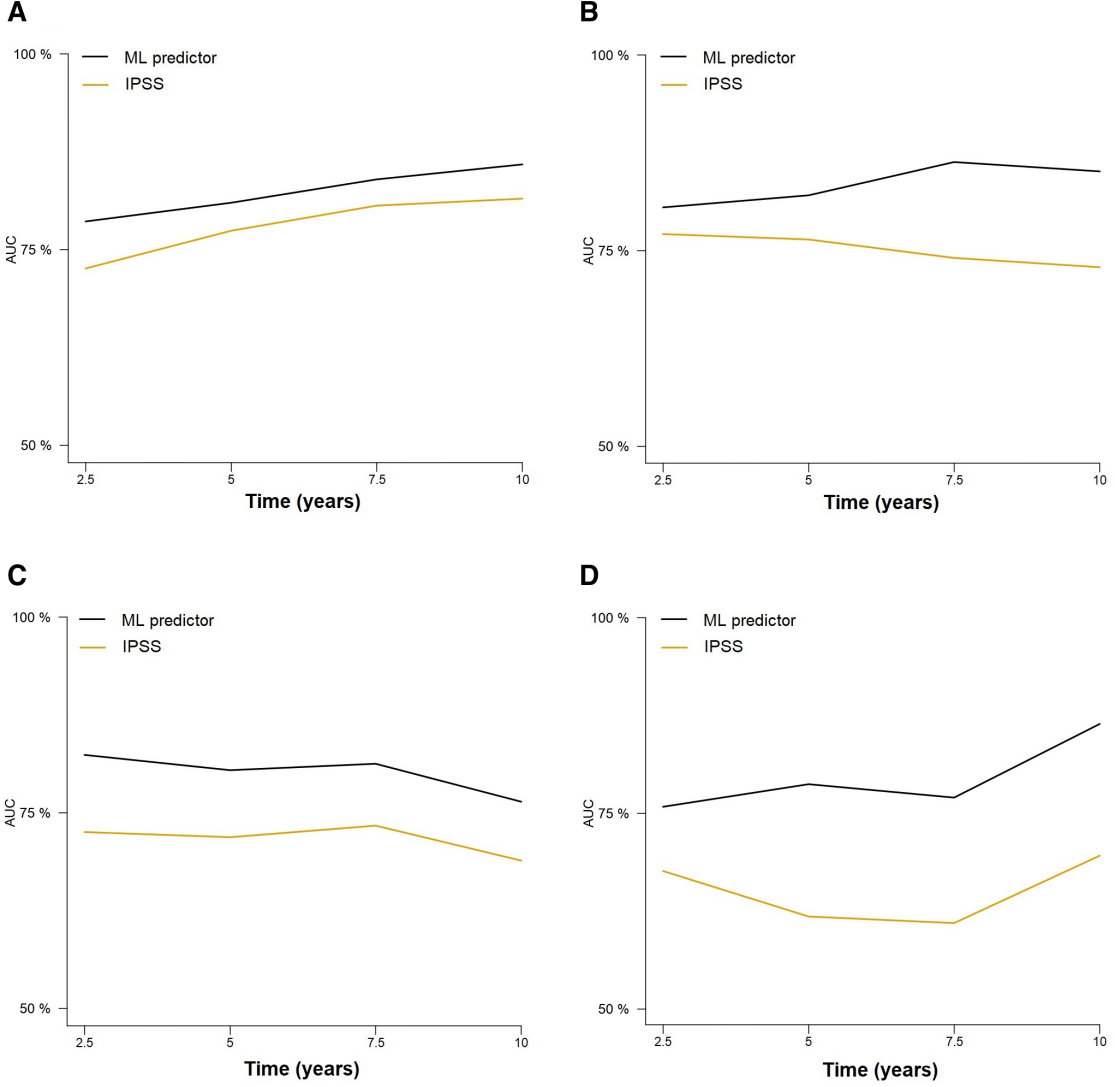
**Performance of the ML model and IPSS score in predicting overall survival in primary myelofibrosis** ([A and B] for the training and test set, respectively) and secondary myelofibrosis ([C and D] for the training and test set, respectively). IPSS = International Prognostic Scoring System, ML = machine learning.

The performance of the ML model was assessed using cross-validated time-dependent AUCs, and a comparison with the IPSS was performed (Suppl. Table S2). The ML model achieved a higher AUC at all evaluated time points (2.5, 5, 7.5, and 10 years) compared with the IPSS, in both the training and test cohorts (Figure 3C and D). The 5- and 10-year AUCs of the ML model were 80.78% and 80.50% in the training set, and 80.61% and 81.95% in the test set. These metrics were superior to those of the IPSS in the same patients: 78.87% and 73.14% in the training cohort, 75.22% and 76.83% in the test set. The difference in performance remained substantial regardless of whether patients were censored or not at time of allo-HCT (Suppl. Figure S1). Next, the IPSS groups were compared with patient risk quartiles according to survival predictions made by the ML algorithm. The distribution of the IPSS groups was unbalanced, with 50.77% and 48.37% of patients being assigned to a lower or higher risk group by the ML algorithm, a fact that particularly affected IPSS intermediate-1 and -2 groups (Figure 4). Furthermore, we were able to confirm the superiority of our model over the IPSS in patients both <60 and ≥60 years (Suppl. Figure S2). Importantly, the model retained a similar prognostic performance in PMF and SMF, whereas the results of the IPSS were poorer in SMF than in PMF (Figure 5). For the present study, PMF patients with grades 0-1 and 2-3 bone marrow fibrosis were considered to have prefibrotic and overt PMF, respectively. The ML performed similarly in both groups, and its prognostic performance was superior to that of the IPSS (Suppl. Figure S3). Finally, the performance of the ML predictor was evaluated in patients within the lower (low, intermediate-1) and higher (intermediate-2, high-risk) IPSS categories. Notably, the performance metrics of the ML classifier were similar regardless of IPSS risk categories (Suppl. Figure S4).

### Comparison of the ML model with the MYSEC-PM in SMF

**Figure 6.**
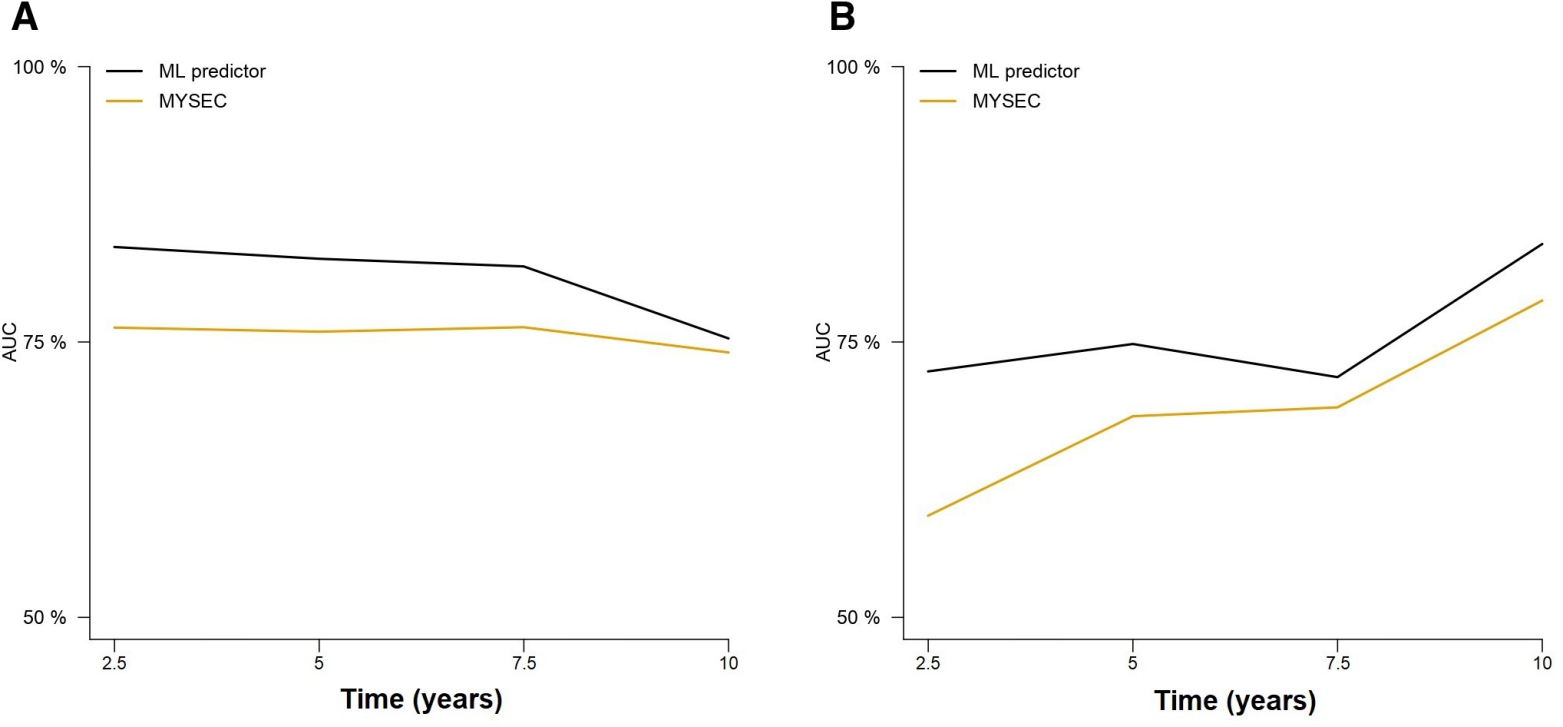
**Comparison of the ML model and the MYSEC-PM model in predicting overall survival in secondary myelofibrosis in the training (A) and test (B) sets.** MYSEC-PM = Myelofibrosis Secondary to PV and ET-Prognostic Model.

We compared the ML predictor with the MYSEC-PM in SMF (Suppl. Table S3; Figure 6). Sufficient information to calculate the MYSEC-PM was available in 449 and 100 SMF patients in the training and test cohorts, respectively. Time-dependent cross-validated AUCs revealed a superior performance of the ML predictor compared to MYSEC-PM in both cohorts. The 5-year AUCs in the training set were 82.55% and 75.93% for the ML model and the MYSEC-PM groups, respectively. Likewise, the 5-year AUCs in the test set were 74.82% for the ML model and 68.26% for the MYSEC-PM.

### Integration of other potential risk factors into the ML model

We evaluated whether other clinically relevant variables collected in a variable number of patients could potentially improve the model. First, we studied the potential benefit of including high-risk cytogenetics in the prediction model. Overall, cytogenetic annotation was available for 277 and 74 cases in the training and test set. Among them, high-risk cytogenetic abnormalities as defined by the DIPSS plus model were present in 23% and 19% of patients in each cohort, respectively. No substantial benefit was observed from the addition of high-risk cytogenetic data to the ML model for the prediction of OS or LFS (Suppl. Tables S4 and S5; Suppl. Figure S5 A-B).

Then, we analyzed the effect of high-risk mutations as defined by the MIPSS70-plus v2.0 (in *ASXL1*, *SRSF2*, *EZH2*, *IDH1*, *IDH2*, and *U2AF1*^*Q157*^ genes^17^) that were detectable with a VAF *≥* 5%. Overall, 247 patients (205 in the training set and 42 in the test set) were fully characterized at the molecular level for high-risk mutations, and patients harboring any of them were considered high-risk for the present analysis. No benefit was obtained by including these molecular abnormalities in the prediction of OS (Suppl. Table S4; Suppl. Figure S6 A-B) or LFS (Suppl. Figure S7; Suppl. Table S5). Next, we assessed the prognostic effect of classifying patients into 4 groups according to MPN driver mutation (*JAK2*-mutated, *CALR*-mutated, *MPL*-mutated, and triple negative). A total of 1138 patients were annotated for this variable, 913 in the training set and 225 in the test set. Nonetheless, no benefit was observed by adding this variable to the ML model (Suppl. Table S4; Suppl. Figure S6 C-D).

We reasoned that the grade of bone marrow fibrosis could play a prognostic role,^16^ but excluded this variable in the initial random forests model due to the different histological classifications of bone marrow fibrosis during the study period and the lack of centralized assessment, taking into account previous reports showing poor concordance in categorizing the degree of fibrosis among pathologists in multicenter studies.^32^ Indeed, univariate analysis revealed that this variable was associated with prognosis in the training and test set (*P* value 2.49 × 10^−7^ and 0.01 in the training and test sets, respectively). However, random forest survival models that included this additional variable did not show improved prognostication (Suppl. Figure S8). Finally, we tested the potential benefit of including Eastern Cooperative Oncology Group (ECOG) performance status^33^ and serum lactate dehydrogenase (LDH) levels at MF diagnosis^34^ in the model. Overall, ECOG was available in 615 patients (483 and 132 cases in the training and test set, respectively) and LDH values were retrieved from 757 patients (602 in the training set and 155 in the test set). None of these variables improved prediction in either cohort when added to the Cox model along with the ML predictor (Suppl. Table S4; Suppl. Figure S9).

### Evaluation of the predictions in tier 2 patients

A total of 231 patients initially excluded from the analysis due to high missing variable rate composed the tier 2 cohort. Baseline characteristics for these patients can be seen in Suppl. Table S6. The missingness rate among the final selected variables was lower than among the original set of evaluated variables (25.16% versus 30.98%). Even so, up to 53.25% of patients had insufficient data for the calculation of the IPSS score. We used parameter imputation in these patients to evaluate the results of the ML prognostic models in this cohort. Using the random forest model for OS prediction, a c-index of 0.730 was obtained. In the case of LFS, the Cox model based on the OS predictors achieved a bootstrapped c-index of 0.606, whereas the random forest model based on the original 8 variables obtained a c-index of 0.654.

**Figure 7.**
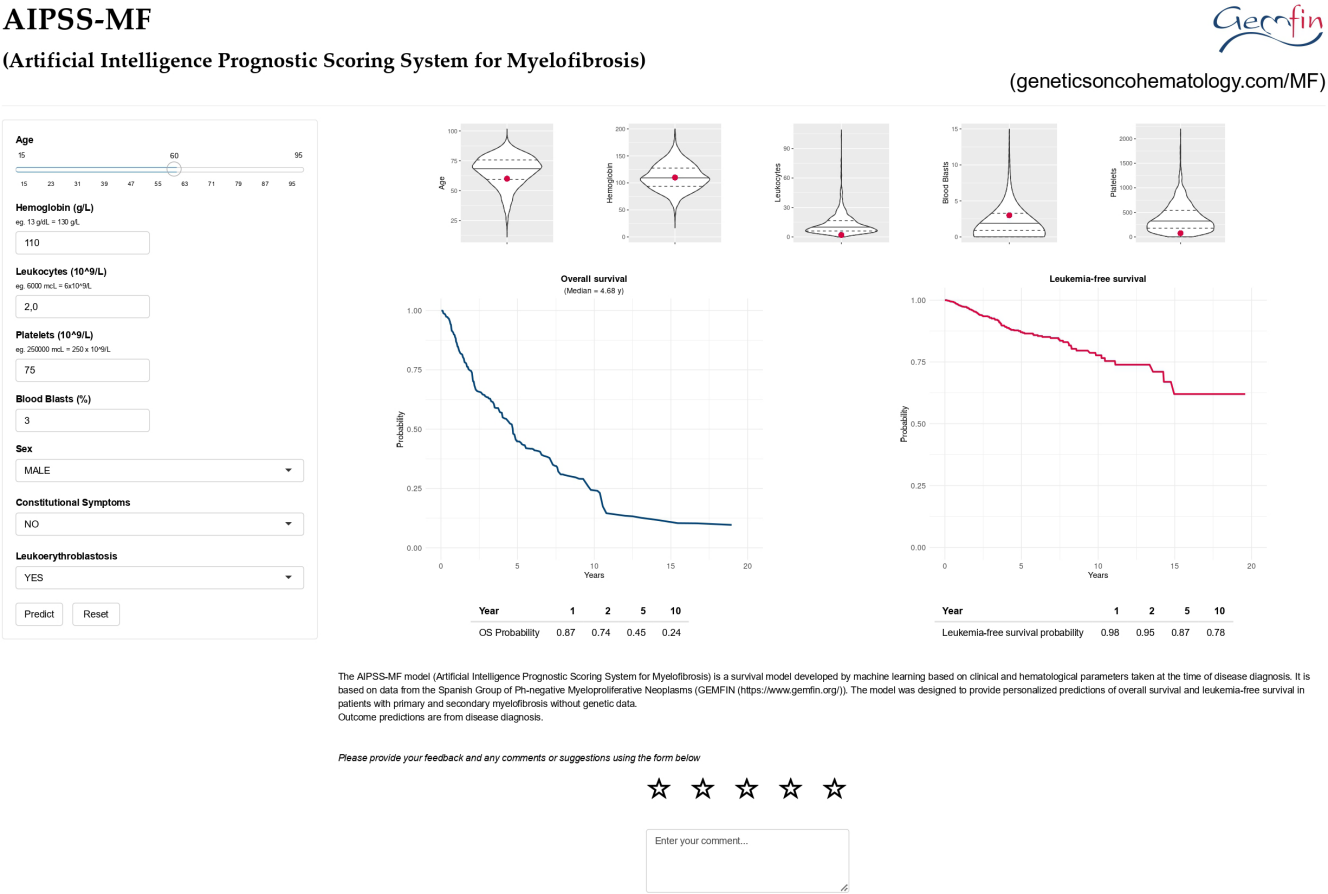
**Use of the interactive online calculator to predict overall and leukemia-free survival in a clinical case.** This is a 60-year-old male patient who presented with fatigue and a palpable spleen 8 cm below the left costal margin. His blood test showed: Hb 11 g/dL, leukocytes 2.0 × 10^9^/L (3% blast cells), platelets 75 × 10^9^/L and leukoerythroblastosis. The patient was assigned to the intermediate-1 risk group by the IPSS model (median predicted survival of 7.9 years). However, the AIPSS-MF model predicted a median survival for this patient of 4.68 years and a leukemia-free survival of 87% at 5 years from diagnosis. As can be seen in the figure, this model is more sensitive than the IPSS to the adverse prognostic features present in patients with the myelodepletive phenotype of myelofibrosis. Unlike the IPSS, the model considers all prognostic information from continuous variables (eg, 3% blood blasts is worse than 1% blood blast, etc.) and does not rely on cutoff points. AIPSS-MF = Artificial Intelligence Prognostic Scoring System for Myelofibrosis; IPSS = International Prognostic Scoring System.

An interactive web calculator of the AIPSS-MF (Artificial Intelligence Prognostic Scoring System for Myelofibrosis) model can be accessed in the following link: https://geneticsoncohematology.com/MF/. The prediction of the model in a clinical case using the interactive calculator is illustrated in Figure 7.

## DISCUSSION

In the present study, we developed an ML model to predict survival in patients with MF based on data from the Spanish Myelofibrosis Registry. The algorithm is a supervised random forest model composed of eight variables obtained at the time of MF diagnosis: age, sex, constitutional symptoms, hemoglobin level, leukocyte count, percentage of blasts in peripheral blood, platelet count, and leukoerytroblastosis. The AIPSS-MF model outperforms currently established prognostic systems, such as the IPSS and MYSEC-PM. In addition, the AIPSS-MF model produces balanced risk groups, reclassifying roughly half of the patients into a different risk category from the IPSS. Other important advantages of the new model are (1) it behaves similarly in PMF and SMF; (2) it provides a personalized risk estimate for each individual patient; and (3) it is not based on genomic data, enabling its implementation in all types of healthcare facilities.

Most of the variables in the new model are among the risk factors included in conventional risk-scoring systems, but here the prognostic impact of continuous variables is not stratified into arbitrary cutoff points. One exception is patient sex, not included in these other models despite the better survival in MF women than men reported in several studies.^20,35^ Likewise, male MPN patients are more prone to acquire high-risk mutations, which have been linked to disease progression.^36^ Anemia has been associated with reduced survival in men, but not in women, and male sex has been associated with a higher risk of leukemic transformation in PMF patients.^37,38^ Finally, in a personalized prognostic model for MPN, male sex has been linked to increased risk of transformation, and mutations in the spliceosome, epigenetic regulators and RAS pathway genes.^19^ To our knowledge, the other factor, leukoerythroblastosis, has not been previously identified as a prognostic risk factor in MF. According to the WHO and ICC classification, this feature is a minor criterion for overt PMF but not for early PMF, and an additional criteria for post-ET MF and post-PV MF, and probably reflects the grade of bone marrow fibrosis.^1,39–41^ In our series, about 60% of patients had leukoerythroblastosis, and this factor correlated with a higher degree of bone marrow fibrosis and a higher frequency of anemia, marked leukocytosis, and circulating blast cells, each of which has been associated with adverse prognosis in MF.^12,14,16,20,41–43^

The main limitations of the present study derive from its registry-based nature. Data quality depends on local physicians entering data at many different centers over a long follow-up period without centralized review. We have considered PMF patients with grades 0-1 and 2-3 bone marrow fibrosis to have prefibrotic and overt PMF, respectively, but the diagnosis of prefibrotic PMF was only formally established in a minority of patients. Therefore, the performance of the AIPSS-MF in patients with prefibrotic PMF requires further validation. Informative cytogenetic data was only available for 25% of patients. The registry includes patients diagnosed from 2000 onwards, and although most (82%) of the series were annotated for MPN driver mutations, only a minority had NGS panel data evaluating additional somatic mutations. Therefore, we were unable to adequately assess the potential benefit of including adverse cytogenetics or high-risk somatic mutations data in our prognostic model. For the same reason, it was not possible to compare the predictive accuracy of our model with those that include genetic information, such as DIPSS plus, MIPSS70 or MIPSS70+ v2.0. The performance of the model was evaluated on a test set comprising 20% of the patients in our registry, but further external evaluation using data from other sources is warranted. Nonetheless, the large size of the patient series, followed over a long observation period, is a principal strength of our study, reflecting the actual clinical course of the entire MF population without the set of inclusion and exclusion criteria used in controlled clinical trials.

The present work contributes further evidence of the profound impact that ML may have in hematology-oncology, reshaping our previous conceptions of disease prognostication and potentially changing our clinical practice. Although ML models are often developed with large datasets that could limit their broad applicability, our results indicate that disease prognostication can be improved by reinterpreting a limited number of classical variables.^44^ Now, more than ever, developing high-quality data banks on patients treated in the real world or in clinical trials will become a defining moment for precision medicine in MF.

In conclusion, we present a new survival model in MF based on data from the Spanish Myelofibrosis Registry. The present model can provide patient-specific predictions at disease diagnosis, and outperforms other well-established risk stratifying systems such as the IPSS and MYSEC-PM. Furthermore, this model is equally predictive of survival in both primary and secondary MF and does not require cytogenetic or molecular data, which facilitates its applicability in all healthcare settings. However, we think that the present model should be used in combination with other models that include genetic information, such as MIPSS70 or MIPSS70+, especially when transplant decisions are involved.

## AUTHOR CONTRIBUTIONS

The GEMFIN group collected the data; AM-O performed the machine learning analysis; JCH-B, MPE, and AM-O wrote the paper; all remaining co-authors critically evaluated the manuscript and made substantial recommendations. All authors approved the submission of this manuscript.

## DISCLOSURES

The Spanish Registry of Myelofibrosis was initially sponsored by a grant from Novartis Pharmaceuticals, Inc. The data supporting the findings of this study are not publicly available due to privacy or ethical restrictions but are available on request from the corresponding authors. The study was approved by the scientific board of GEMFIN. The authors have no conflicts of interest to disclose.

## Supplementary Material
