# Supplementary material for: Machine Learning Improves Risk Stratification in Myelofibrosis: An Analysis of the Spanish Registry of Myelofibrosis
Source: Hemasphere. 2022 Dec 20;7(1):e818. doi: 10.1097/HS9.0000000000000818 (PMC9771324; doi:10.1097/HS9.0000000000000818)
Supplement: Supplementary file 1 [file hs9-7-e818-s001.docx]

# Supplemental data

**Machine learning improves risk stratification in myelofibrosis:**

**an analysis of the Spanish Registry of Myelofibrosis**

| Supplementary Table 1. Time-dependent AUCs for the ML and IPSS models in the prediction of AML transformation | | | | | | | | |
| --- | --- | --- | --- | --- | --- | --- | --- | --- |
|  | **2.5 years** | | **5 years** | | **7.5 years** | | **10 years** | |
|  | **Training set** | **Test set** | **Training set** | **Test set** | **Training set** | **Test set** | **Training set** | **Test set** |
| **ML model** | 0.744 | 0.757 | 0.753 | 0.784 | 0.791 | 0.800 | 0.796 | 0.844 |
| **IPSS** | 0.686 | 0.706 | 0.718 | 0.666 | 0.752 | 0.656 | 0.781 | 0.753 |
|  |  |  |  |  |  |  |  |  |

| Supplementary Table 2. Time-dependent AUCs for predicting overall survival with the ML and IPSS models in the whole cohort, as well as in different patient subgroups | | | | | | | | |
| --- | --- | --- | --- | --- | --- | --- | --- | --- |
|  | **2.5 years** | | **5 years** | | **7.5 years** | | **10 years** | |
|  | **Training set** | **Test set** | **Training set** | **Test set** | **Training set** | **Test set** | **Training set** | **Test set** |
| **ML whole cohort** | 0.797 | 0.778 | 0.806 | 0.808 | 0.829 | 0.835 | 0.820 | 0.855 |
| **IPSS whole cohort** | 0.724 | 0.752 | 0.752 | 0.729 | 0.777 | 0.713 | 0.768 | 0.731 |
| **ML whole cohort (censored at transplant)** | 0.760 | 0.755 | 0.717 | 0.770 | 0.755 | 0.812 | 0.730 | 0.834 |
| **IPSS whole cohort (censored at transplant)** | 0.696 | 0.734 | 0.729 | 0.706 | 0.765 | 0.700 | 0.752 | 0.727 |
| **ML ≥ 60 years** | 0.773 | 0.784 | 0.772 | 0.766 | 0.787 | 0.812 | 0.782 | 0.800 |
| **IPSS ≥ 60 years** | 0.676 | 0.713 | 0.703 | 0.635 | 0.736 | 0.625 | 0.741 | 0.623 |
| **ML < 60 years** | 0.860 | 0.850 | 0.785 | 0.884 | 0.775 | 0.760 | 0.743 | 0.772 |
| **IPSS < 60 years** | 0.795 | 0.825 | 0.738 | 0, 835 | 0.719 | 0.653 | 0.669 | 0.656 |
| **ML: Primary MF** | 0.786 | 0.805 | 0.810 | 0.821 | 0.840 | 0.863 | 0.859 | 0.851 |
| **IPSS: Primary MF** | 0.726 | 0.771 | 0.774 | 0.764 | 0.806 | 0.741 | 0.815 | 0.729 |
| **ML: Secondary MF** | 0.824 | 0.758 | 0.804 | 0.787 | 0.813 | 0.770 | 0.764 | 0.864 |
| **IPSS: Secondary MF** | 0.725 | 0.676 | 0.719 | 0.618 | 0.734 | 0.610 | 0.689 | 0.696 |
| **ML: IPSS low risk/intermediate-1** | 0.854 | 0.500 | 0.765 | 0.788 | 0.826 | 0.925 | 0.732 | 0.882 |
| **ML: IPSS intermediate-2/high risk** | 0.776 | 0.750 | 0.781 | 0.769 | 0.795 | 0.802 | 0.792 | 0.832 |

| Supplementary Table 3. Time-dependent AUCs for the ML predictions vs MYSEC-PM scores in patients with secondary MF. Comparisons were performed with risk groups and points estimated using both scores. | | | | | | | | |
| --- | --- | --- | --- | --- | --- | --- | --- | --- |
|  | **2.5 years** | | **5 years** | | **7.5 years** | | **10 years** | |
|  | **Training set** | **Test set** | **Training set** | **Test set** | **Training set** | **Test set** | **Training set** | **Test set** |
| **3. ML model** | 0.836 | 0.723 | 0.826 | 0.748 | 0.819 | 0.718 | 0.753 | 0.839 |
| **3. MYSEC-PM groups** | 0.763 | 0.592 | 0.759 | 0.683 | 0.763 | 0.691 | 0.740 | 0.788 |
| **4. ML model** | 0.836 | 0.733 | 0.829 | 0.767 | 0.827 | 0.737 | 0.767 | 0.846 |
| **4. MYSEC-PM points** | 0.787 | 0.662 | 0.807 | 0.700 | 0.820 | 0.673 | 0.775 | 0.741 |

| Supplementary Table 4. Time-dependent AUCs for predicting overall survival using the ML predictions alone and in combination with different prognostic variables | | | | | | | | |
| --- | --- | --- | --- | --- | --- | --- | --- | --- |
|  | **2.5 years** | | **5 years** | | **7.5 years** | | **10 years** | |
|  | **Training set** | **Test set** | **Training set** | **Test set** | **Training set** | **Test set** | **Training set** | **Test set** |
| **1. ML model** | 0.823 | 0.738 | 0.807 | 0.766 | 0.839 | 0.838 | 0.855 | 0.910 |
| **1. ML model + LDH** | 0.823 | 0.729 | 0.805 | 0.760 | 0.835 | 0.827 | 0.856 | 0.894 |
| **2. ML model** | 0.808 | 0.755 | 0.802 | 0.754 | 0.840 | 0.836 | 0.828 | 0.848 |
| **2. ML model + ECOG** | 0.801 | 0.757 | 0.801 | 0.749 | 0.836 | 0.826 | 0.815 | 0.842 |
| **3. ML model** | 0.813 | 0.757 | 0.824 | 0.809 | 0.836 | 0.824 | 0.805 | 0.860 |
| **3. ML model + Driver Mutations** | 0.813 | 0.760 | 0.826 | 0.810 | 0.838 | 0.829 | 0.798 | 0.849 |
| **4. ML model** | 0.817 | 0.864 | 0.834 | 0.837 | 0.796 | 0.855 | 0.788 | 0.950 |
| **4. ML model + High Risk Mutations** | 0.793 | 0.854 | 0.822 | 0.853 | 0.796 | 0.787 | 0.796 | 0.842 |
| **5. ML model** | 0. 790 | 0. 810 | 0. 794 | 0. 778 | 0. 842 | 0. 802 | 0. 810 | 0. 890 |
| **5. ML model + High Risk Cytogenetics** | 0. 797 | 0. 796 | 0. 811 | 0. 765 | 0. 844 | 0. 787 | 0. 805 | 0. 880 |

| Supplementary Table 5. Time-dependent AUCs for the ML predictions alone or combined with high-risk mutations or adverse cytogenetics in the prediction of time to AML evolution. | | | | | | | | |
| --- | --- | --- | --- | --- | --- | --- | --- | --- |
|  | **2.5 years** | | **5 years** | | **7.5 years** | | **10 years** | |
|  | **Training set** | **Test set** | **Training set** | **Test set** | **Training set** | **Test set** | **Training set** | **Test set** |
| **1. ML model** | 0.777 | 0.771 | 0.772 | 0.810 | 0.725 | 0.817 | 0.721 | 0.954 |
| **1. ML model + High Risk Mutations** | 0.717 | 0.791 | 0.705 | 0.690 | 0.730 | 0.701 | 0.734 | 0.778 |
| **2. ML model** | 0. 716 | 0. 822 | 0. 821 | 0. 728 | 0. 837 | 0. 776 | 0. 838 | 0. 869 |
| **2. ML model + High Risk Cytogenetics** | 0. 719 | 0. 813 | 0. 824 | 0. 688 | 0. 861 | 0. 706 | 0. 838 | 0. 782 |

| Supplementary Table 6. Baseline characteristics of tier 2 patients. | |
| --- | --- |
| **Variable** | **Training** |
| N | 231 |
| Age*, years | 70.64 (29.35-93.10) |
| Age > 65 years, % | 66.67 |
| Male/Female sex, % | 63.63 / 36.37 |
| Constitutional symptoms, % | 35.19 |
| Leukocyte counts*, x 10^9^/L | 10.1 (1.3-148) |
| Leukocytes >25 x 10^9^/L, % | 12.64 |
| Hemoglobin level*, g/dL | 110 (58-172) |
| Hemoglobin < 10 g/dL, % | 33.89 |
| Platelet count*, x 10^9^/L | 325 (3-1668) |
| Platelets < 100 x 10^9^/L, % | 15.82 |
| Blood blasts count**, % | 0.91 (0-9) |
| Blood blasts ≥1%, % | 24.24 |
| Leukoerythroblastosis, % | 52.67 |
| Median follow-up, years | 7.31 (4.73-11.73) |
| Median overall survival (95% CI), years | 5.34 (4.81-6.52) |
| Progression to AML, % | 14.71 |
| *Median (range) |  |
| **Mean (range) |  |

**Supplementary Figure 1.** Time-dependent cross-validated AUCs in the training (A) and test (B) cohorts for predicting overall survival according to the ML model and the IPSS score. For this analysis, survival was censored at the time of transplantation.


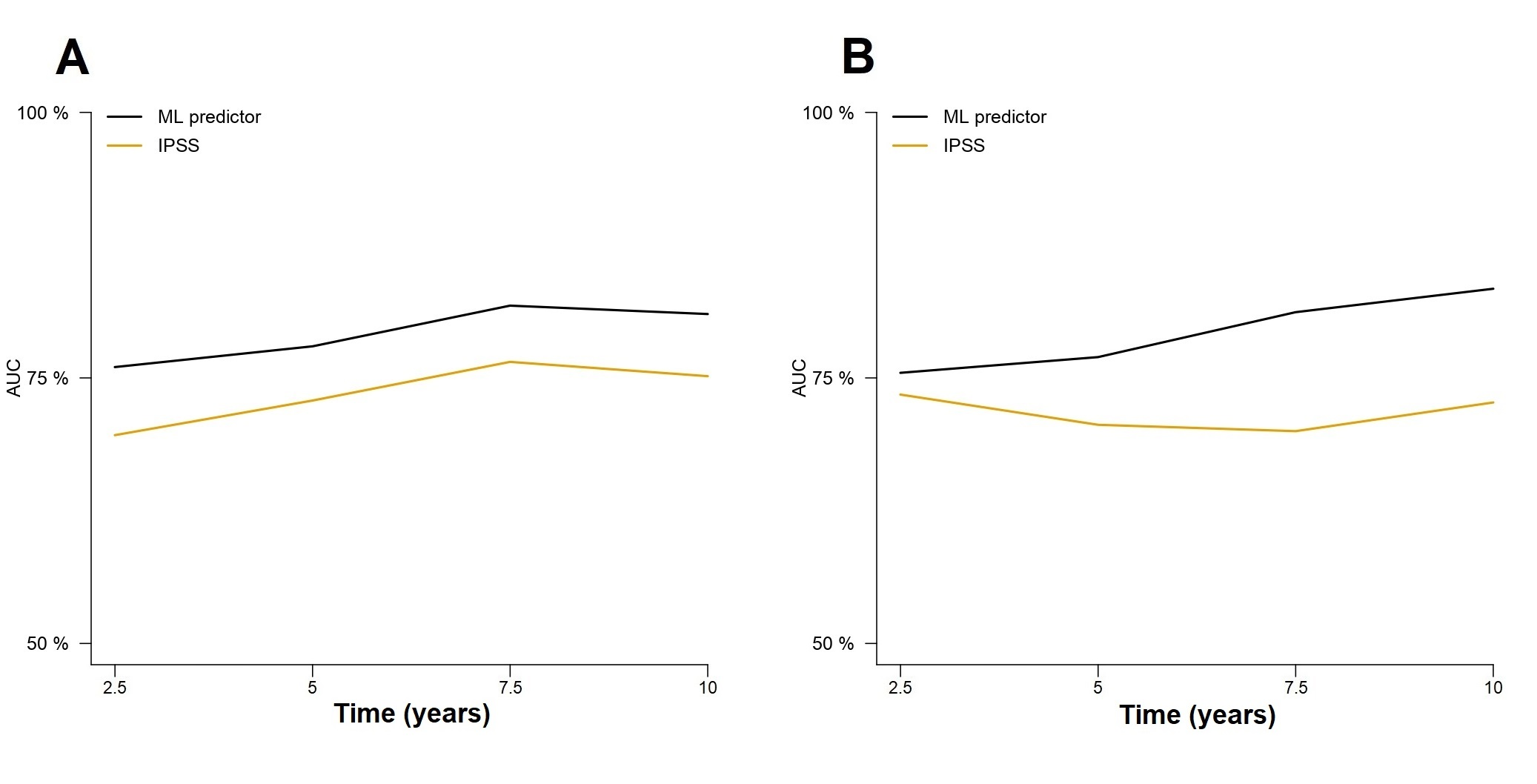


**Supplementary Figure 2.** Time-dependent cross-validated AUCs for the prediction of overall survival in patients <60 years and >60 years. Panels A and B represent comparisons of the ML model and the IPSS in patients <60 years for the training and test set, respectively, whereas panels C and D represent comparisons in the training and test set cohorts, respectively, in patients ≥60 years.


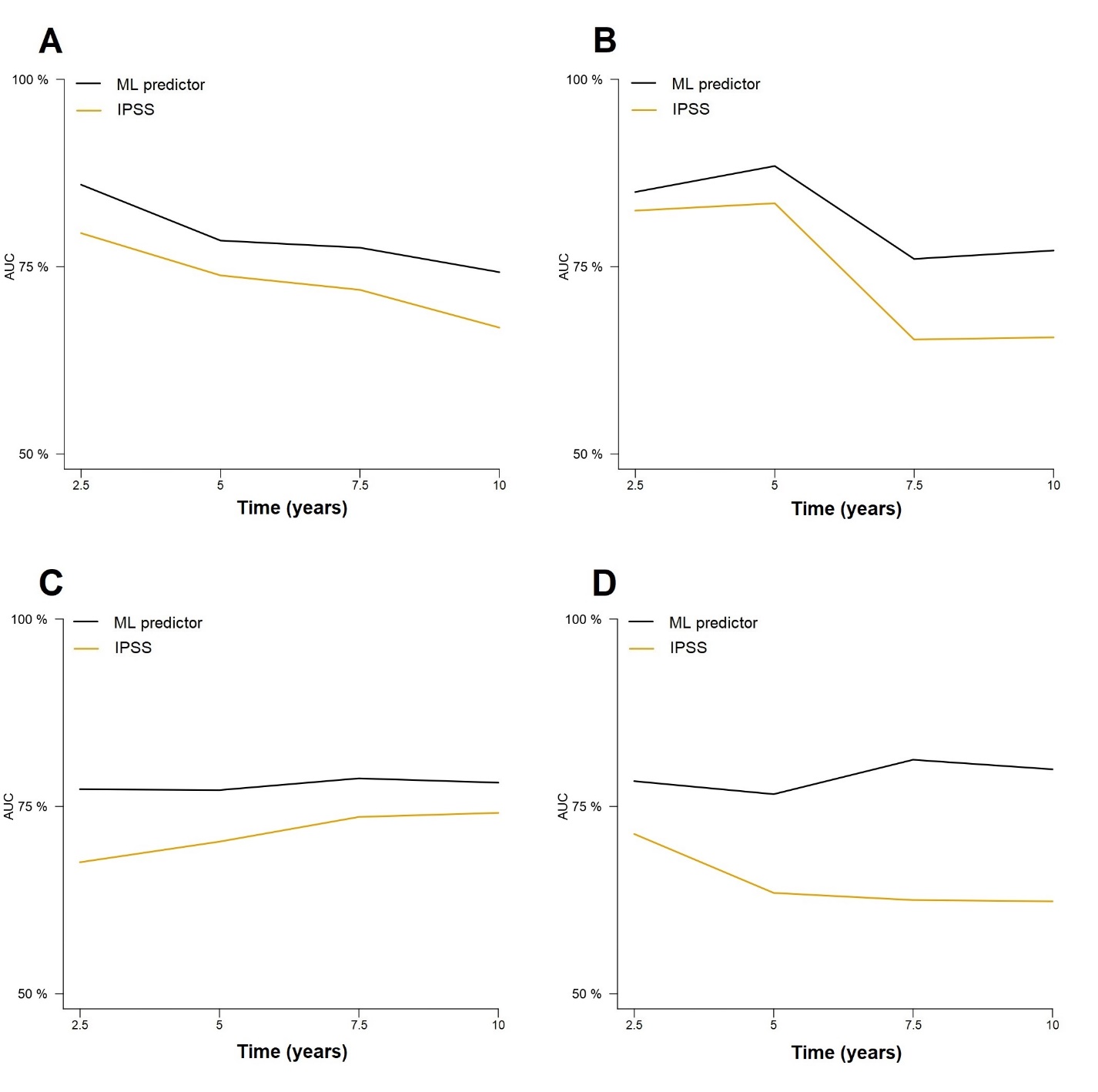


**Supplementary Figure 3.** Performance of the ML model and IPSS in predicting overall survival in patients with prefibrotic or overt PMF in the training set (A and B panels). Performance of the ML model and IPSS in predicting overall survival in patients with overt PMF in the test set (C). Cross-validated estimates of patients with prefibrotic PMF in the test set were not extracted due to limited sample size (N=34).


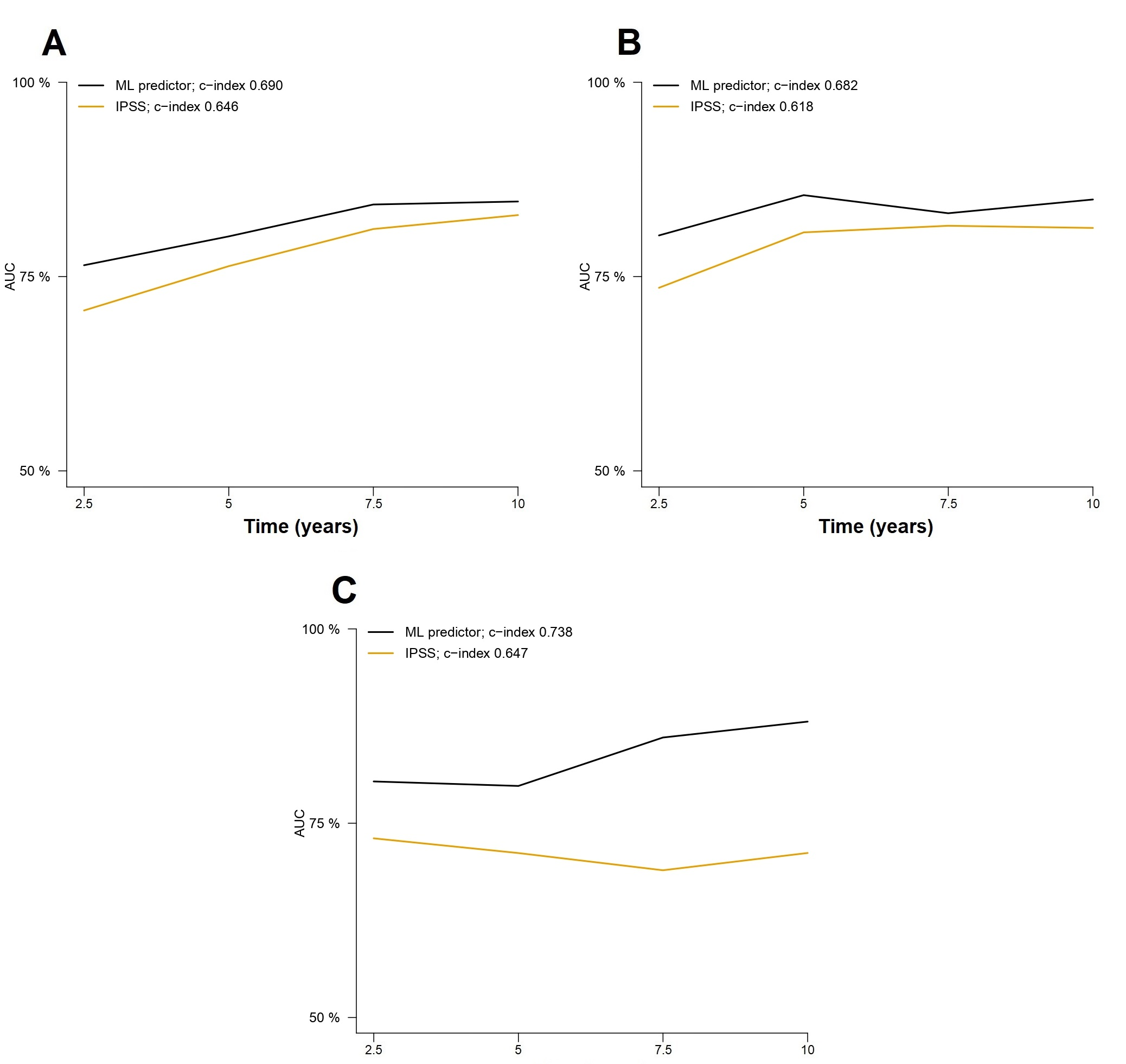


**Supplementary Figure 4.** Prognostic performance of the ML model in patients within the lower risk IPSS categories (low and intermediate 1, 0-1 points) in the training (A) and test set (B). Panels C and D report the results of the same analysis for patients in the higher risk IPSS categories (intermediate-2 and high risk, 2-3 points).


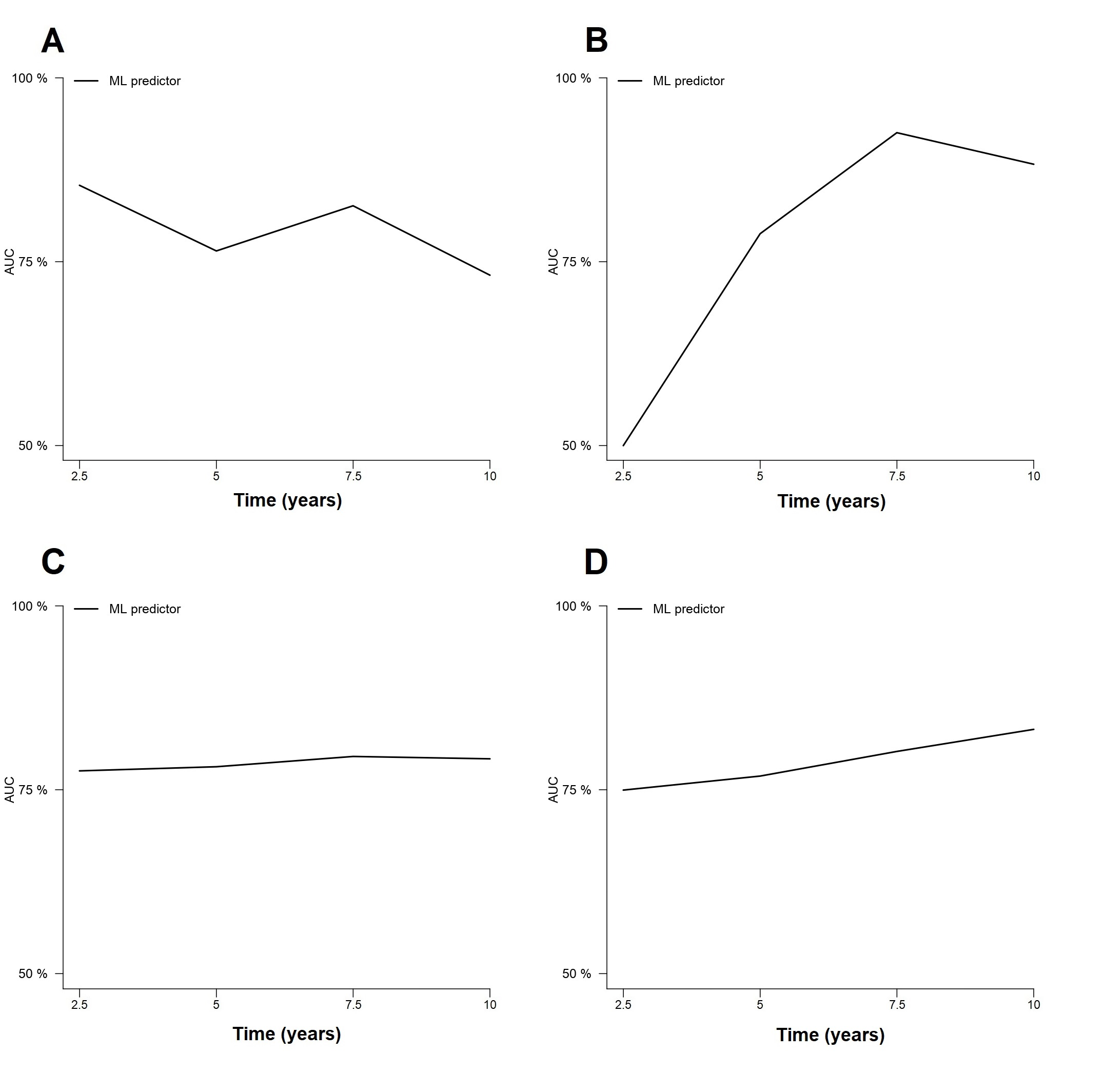


**Supplementary Figure 5.** Comparison of the ML model predictions without and with high-risk cytogenetics in the training (A) and test (B) sets.


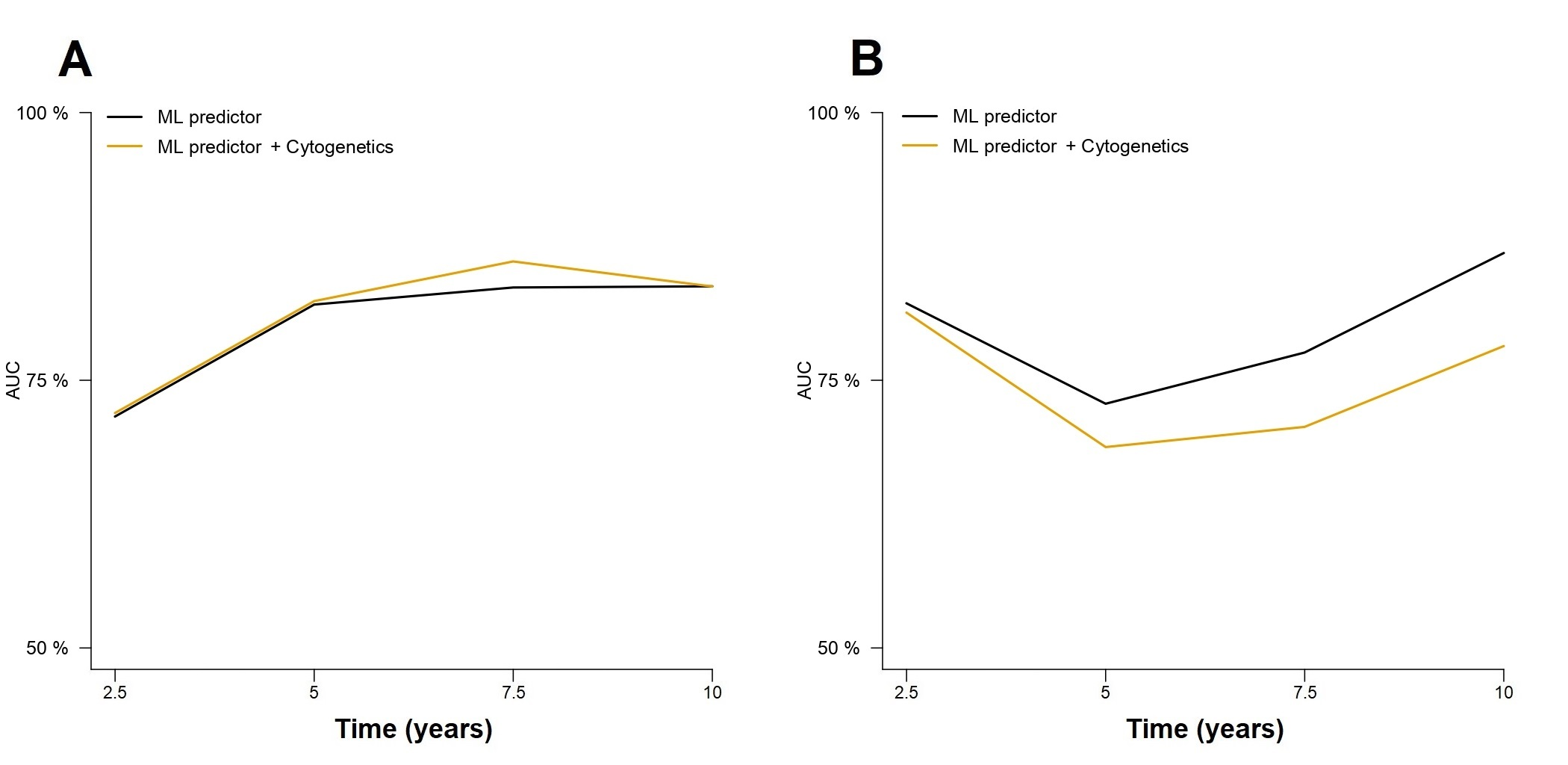


**Supplementary Figure 6.** Comparison of the ML model predictions without and with high-risk mutations in the training (A) and test (B) sets. C-D) Comparison of the ML model predictions without and with driver mutation genotypes (*JAK2*, *CALR*, *MPL* and triple negative) in the training (C) and test (D) sets.


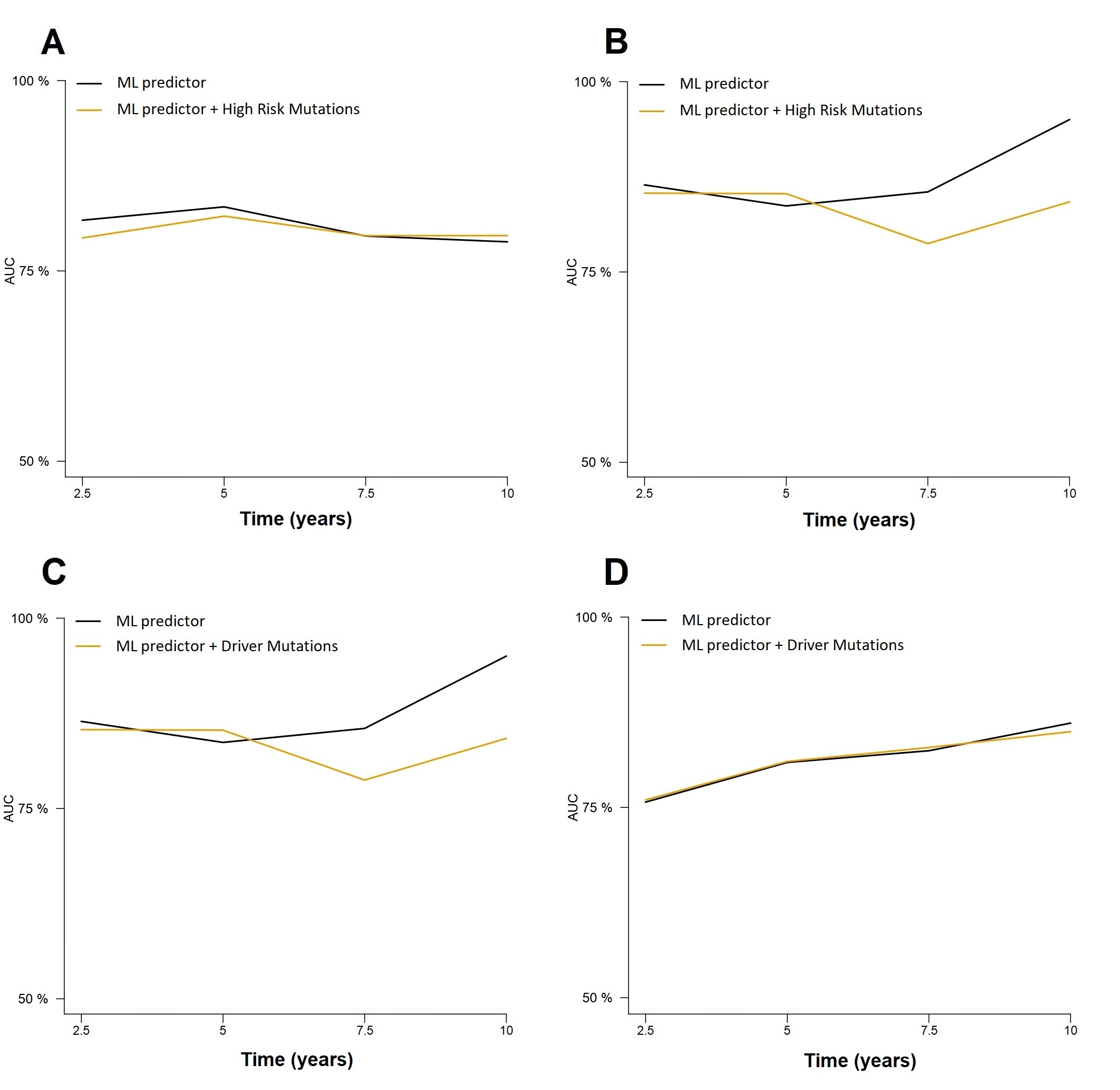


**Supplementary Figure 7.** A-B) Comparison of the ML model predictions without and with high-risk mutations for the prediction of leukemia-free survival in the training (A) and test (B) sets.


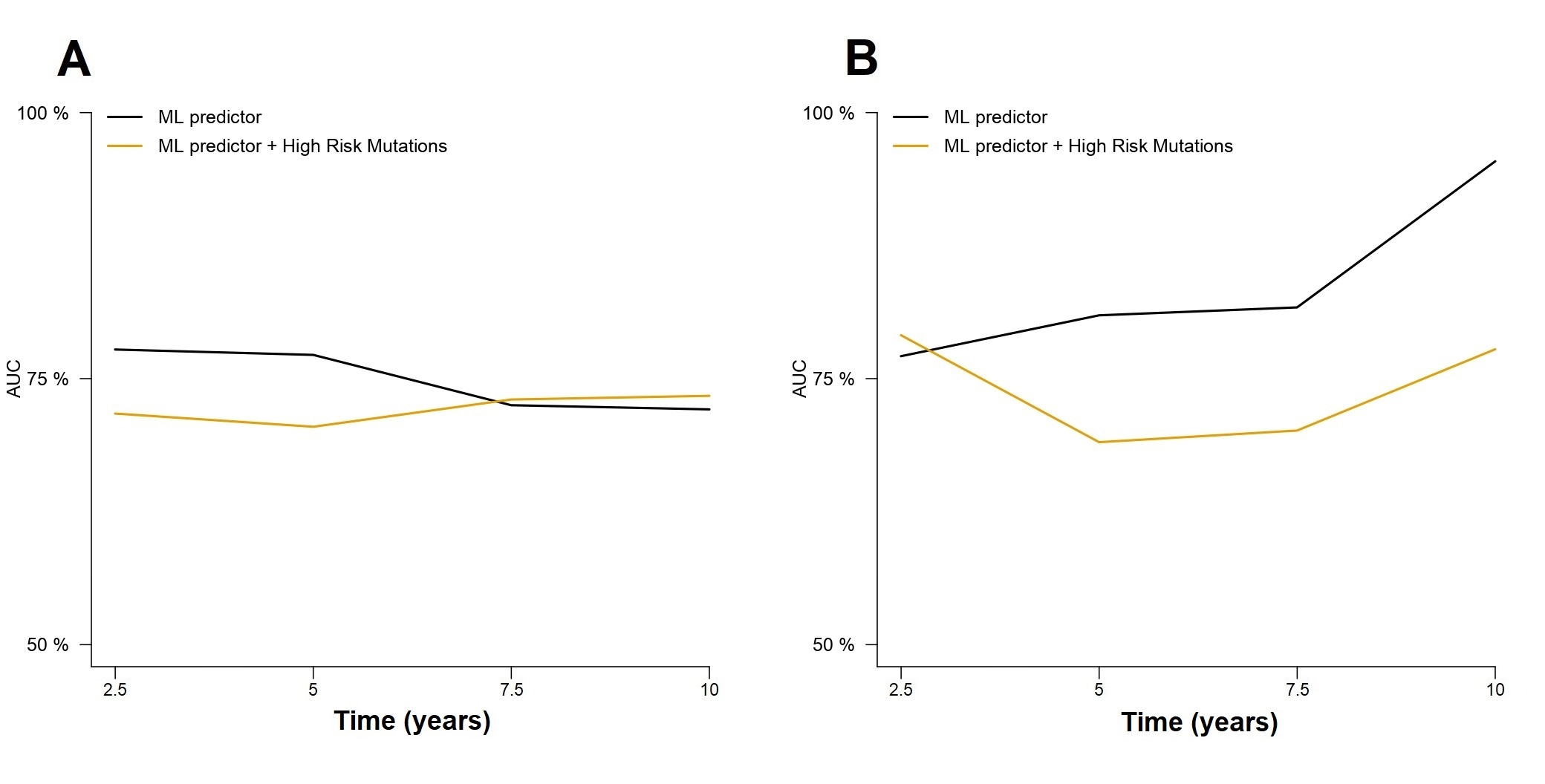


**Supplementary Figure 8.** Comparison of the ML model predictions of overall survival considering or not bone marrow fibrosis grade according to WHO classification in the training (A) and test (B) sets.


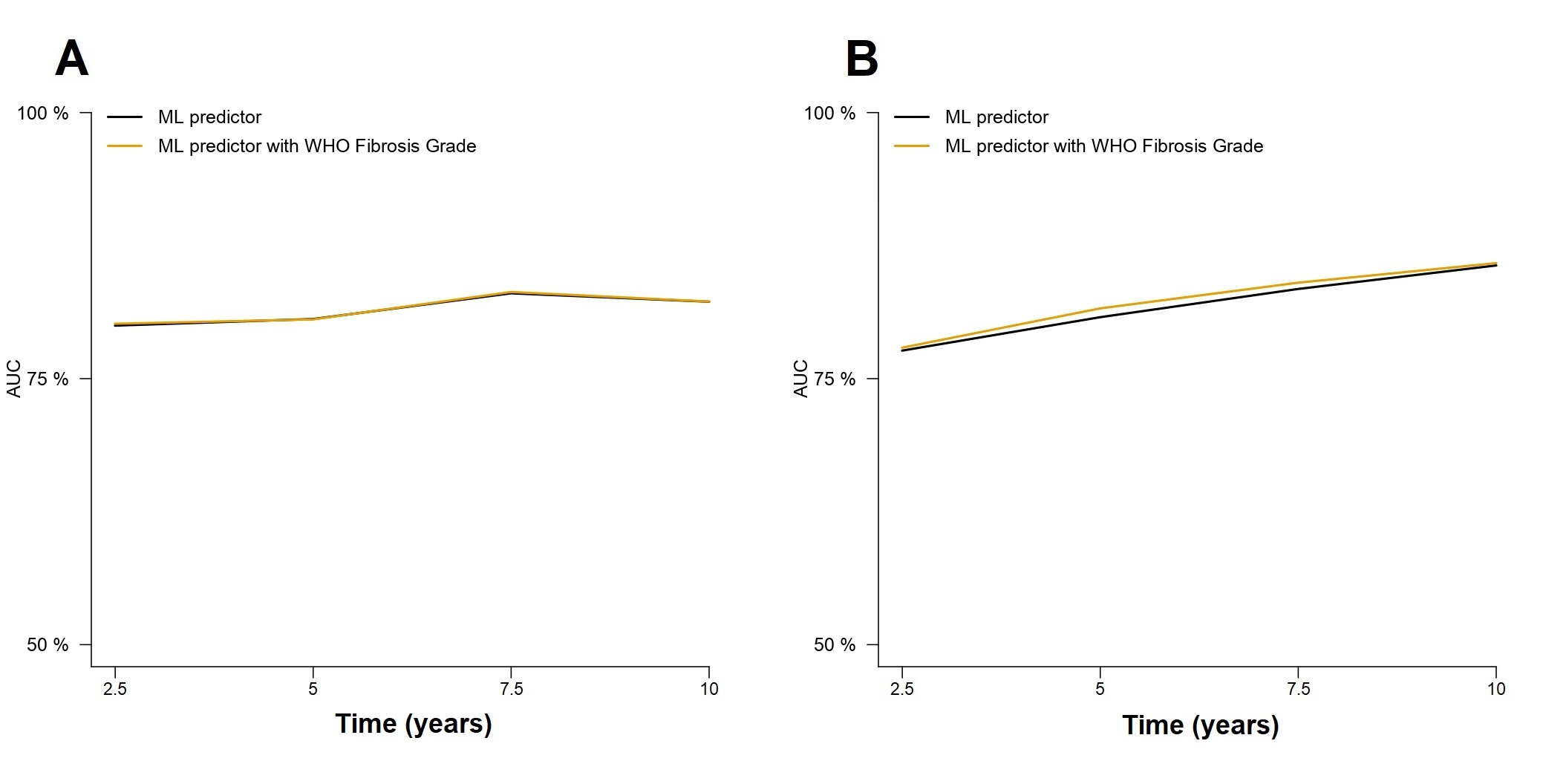


**Supplementary Figure 9.** A-B) Comparison of the ML model predictions without and with serum LDH values in the training (A) and test (B) sets. C-D) Comparison of ML model predictions without and with ECOG score in the training (C) and test (D) sets.


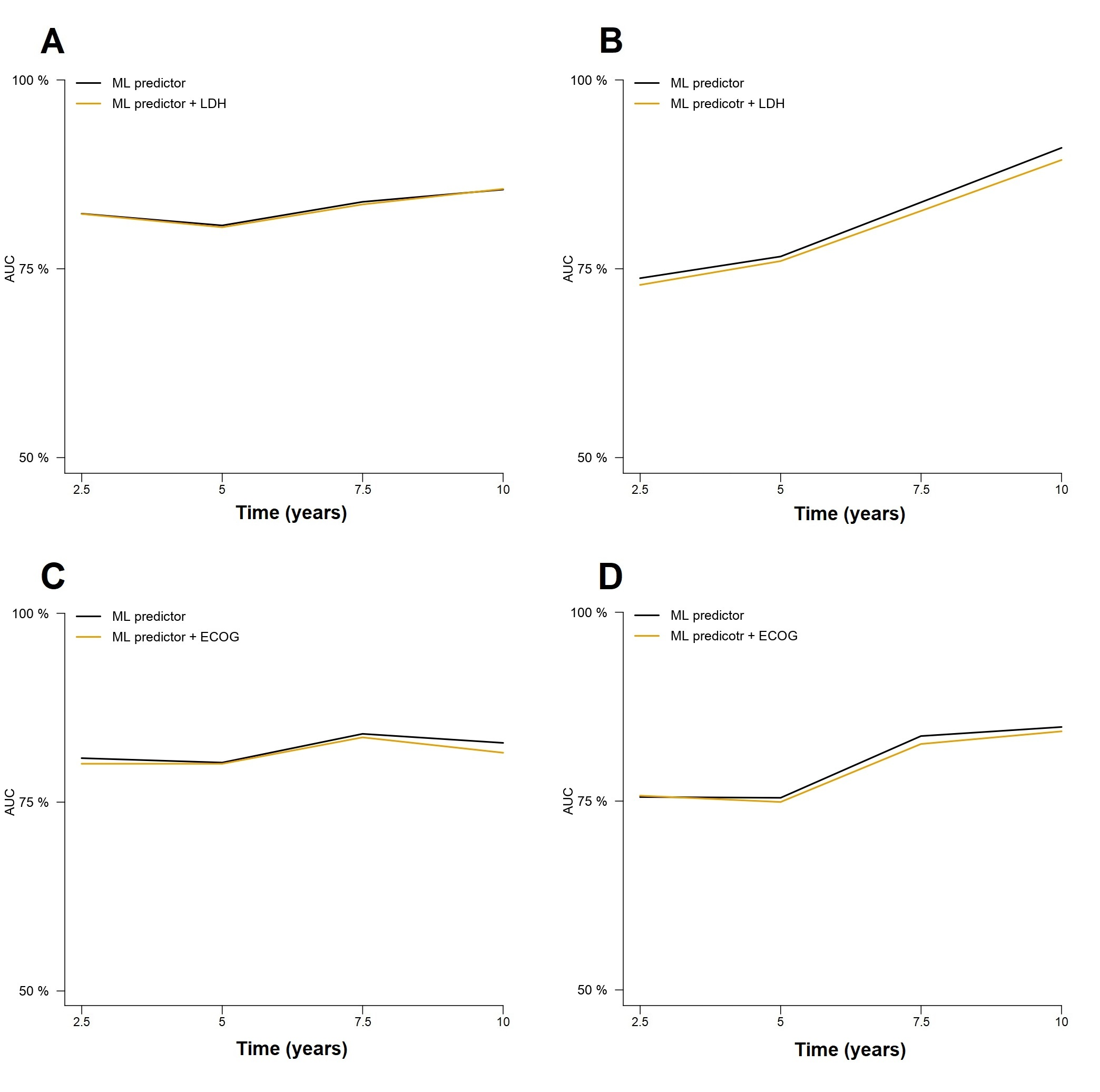


# GEMFIN centers contributing to the Spanish Myelofibrosis Registry

Mª Isabel Mata Vázquez, COMPLEJO HOSPITAL COSTA DEL SOL; María Luisa Antelo Caamaño, COMPLEJO HOSPITALARIO DE NAVARRA; María Concepción Ruíz Nuño, COMPLEJO HOSPITALARIO REGIONAL DE MÁLAGA; Carlos Fernández Lago, COMPLEXO HOSPITALARIO UNIVERSITARIO A CORUÑA; Manuel Pérez Encinas, COMPLEXO HOSPITALARIO UNIVERSITARIO DE SANTIAGO DE COMPOSTELA; María José Lis, Francisco Ibáñez Alís, CONSORCIO HOSPITAL GENERAL UNIVERSITARIO DE VALENCIA; María Dolores Carrera Merino, HOSPITAL ARNAU DE VILANOVA DE VALENCIA; Alberto Álvarez Larrán, Eduardo Arellano, HOSPITAL CLINIC I PROVINCIAL DE BARCELONA; Asunción Peña, HOSPITAL CLÍNICO SAN CARLOS; Ana García Bacelor, HOSPITAL CLINICO UNIVERSITARIO DE VALLADOLID; José Antonio Moreno Chulilla, HOSPITAL CLÍNICO UNIVERSITARIO LOZANO BLESA; Juan Carlos Hernández Boluda, HOSPITAL CLÍNICO UNIVERSITARIO VALENCIA; Raúl Pérez López, HOSPITAL CLÍNICO UNIVERSITARIO VIRGEN DE LA ARRIXACA; Mª José Ramírez, HOSPITAL DE JEREZ DE LA FRONTERA; María Teresa Gómez Casares, María del Mar Perera Álvarez, María Nieves Sáez Perdomo, HOSPITAL DE GRAN CANARIA DR. NEGRIN; Clara Martínez Valverde, Silvana Saavedra Gerosa, HOSPITAL DE LA SANTA CREU I SANT PAU; Natalia De Heras Rodríguez, HOSPITAL DE LEÓN; Patricia Vélez Tenza, HOSPITAL DEL MAR; Dolors Vela Payan, Montserrat Cortés Sansa, HOSPITAL GENERAL DE GRANOLLERS; Carmen Olivier, Virginia Cardos Gómez, HOSPITAL GENERAL DE SEGOVIA; Carmen García Hernández, HOSPITAL GENERAL UNIVERSITARIO DE ALICANTE; Santiago Osorio, HOSPITAL GENERAL UNIVERSITARIO GREGORIO MARAÑÓN; Francisca Ferrer Marín, Raquel Pla García, HOSPITAL MORALES MESEGUER; Valentín García Gutiérrez, María Casanova, HOSPITAL RAMÓN Y CAJAL; José Ramón Molina, HOSPITAL REINA SOFÍA; José María Alonso Alonso, Lucía Guerrero Fernández, HOSPITAL RIO CARRIÓN; Ángela Martínez Hellín, HOSPITAL SAN CECILIO; Ana Belén Dueñas Pérez, HOSPITAL SANTA BÁRBARA; Armando Luaña Galán, HOSPITAL UNIVERSITARI ARNAU DE VILANOVA DE LLEIDA; Anna Angona, Miguel Sagüés Serrano, Berta Valls, HOSPITAL UNIVERSITARI DE GIRONA DR. JOSEP TRUETA; Blanca Xicoy Cirici, HOSPITAL UNIVERSITARI GERMANS TRIAS I PUJOL DE BADALONA; Elvira Mora, Silvia García Palomares, HOSPITAL UNIVERSITARI I POLITÈCNIC LA FE; Janilson Do Nascimiento Ferreira, HOSPITAL UNIVERSITARI JOAN XXIII DE TARRAGONA; María Antonia Durán Pastor, HOSPITAL UNIVERSITARI SON ESPASES; Laura Fox, HOSPITAL UNIVERSITARI VALL D''HEBRON; Rosa María Ayala Díaz, Gonzalo Carreño, HOSPITAL UNIVERSITARIO 12 DE OCTUBRE; Ángel Ramírez Páyer, HOSPITAL UNIVERSITARIO CENTRAL DE ASTURIAS; Beatriz Cuevas, HOSPITAL UNIVERSITARIO DE BURGOS; José María Raya Sánchez, Marta Fernández González, HOSPITAL UNIVERSITARIO DE CANARIAS (H.U.C); Pilar Aragües, Miriam Vara, HOSPITAL UNIVERSITARIO DE CRUCES; Nieves Somolinos de Marcos, HOSPITAL UNIVERSITARIO DE GETAFE; Gabriela Silva Carreras, Juan Luis Steegmann Olmedillas, HOSPITAL UNIVERSITARIO DE LA PRINCESA; Jesús Mª Hernández Rivas, Alejandro Avendaño Pita, María Luisa Martín Mateos, HOSPITAL UNIVERSITARIO DE SALAMANCA; Elvira Gómez Sanz, M. Teresa Cobo, HOSPITAL UNIVERSITARIO DEL SURESTE; María José Fernández Llavador, HOSPITAL UNIVERSITARIO DR. PESET; Juan Manuel Alonso Domínguez, HOSPITAL UNIVERSITARIO FUNDACIÓN JIMÉNEZ DÍAZ; José Ángel Hernández Rivas, María Ángeles Foncillas, HOSPITAL UNIVERSITARIO INFANTA LEONOR; Raúl Córdoba Mascuñano, MarÍa José Penalva, HOSPITAL UNIVERSITARIO INFANTA SOFÍA; Ana Esther Kerguelen Fuentes, Mercedes Gasior Kabat, HOSPITAL UNIVERSITARIO LA PAZ; Sonia González de Villambrosia, Miguel Ángel Cortés Vázquez, HOSPITAL UNIVERSITARIO MARQUÉS DE VALDECILLA; Gonzalo Caballero, HOSPITAL UNIVERSITARIO MIGUEL SERVET; Elena Magro Mazo, HOSPITAL UNIVERSITARIO PRÍNCIPE DE ASTURIAS; Alberto Cantalapiedra Díez, Luis Javier García Frade, HOSPITAL UNIVERSITARIO RIO HORTEGA; Francisca Mª Hernández Mohedo, HOSPITAL UNIVERSITARIO VIRGEN DE LAS NIEVES; María García Fortés, Regina García Delgado, HOSPITAL VIRGEN DE LA VICTORIA; Elisa Arbelo Granados, HOSPITAL VIRGEN MACARENA; Concepción Boqué Genovard, María Alicia Senín, INSTITUT CATALÀ D'ONCOLOGIA L'HOSPITALET (ICO); Erik De Cabo López, HOSPITAL EL BIERZO; Magdalena Sierra Pacho, HOSPITAL VIRGEN DE LA CONCHA; José Antonio González, HOSPITAL VIRGEN DEL PUERTO.

**List of patients included in the study in each center**

| **Center code** | **No of patients included** |
| --- | --- |
| 1 | 25 |
| 2 | 51 |
| 3 | 5 |
| 4 | 3 |
| 5 | 70 |
| 6 | 26 |
| 7 | 52 |
| 8 | 92 |
| 9 | 13 |
| 10 | 50 |
| 11 | 25 |
| 12 | 26 |
| 13 | 30 |
| 14 | 36 |
| 15 | 79 |
| 16 | 20 |
| 17 | 7 |
| 18 | 43 |
| 19 | 44 |
| 21 | 15 |
| 23 | 13 |
| 24 | 13 |
| 25 | 33 |
| 26 | 30 |
| 27 | 18 |
| 28 | 23 |
| 29 | 40 |
| 30 | 5 |
| 31 | 12 |
| 32 | 42 |
| 33 | 53 |
| 34 | 4 |
| 35 | 6 |
| 36 | 26 |
| 37 | 15 |
| 38 | 46 |
| 39 | 13 |
| 40 | 68 |
| 41 | 31 |
| 42 | 11 |
| 200 | 18 |
| 202 | 33 |
| 202 | 3 |
| 203 | 15 |
| 204 | 9 |
| 205 | 174 |
| 206 | 78 |
| 209 | 2 |
| 210 | 9 |
| 211 | 8 |
| 212 | 4 |
| 213 | 15 |
| 215 | 10 |
| 216 | 5 |
| 225 | 8 |
| 226 | 2 |
| 227 | 1 |
| 228 | 3 |
| 229 | 4 |
| 230 | 2 |
| **Total** | **1617** |
